# Strategies and Solutions for Team Sports Athletes in Isolation due to COVID-19

**DOI:** 10.3390/sports8040056

**Published:** 2020-04-24

**Authors:** Igor Jukic, Julio Calleja-González, Francesc Cos, Francesco Cuzzolin, Jesús Olmo, Nicolas Terrados, Nenad Njaradi, Roberto Sassi, Bernardo Requena, Luka Milanovic, Ivan Krakan, Kostas Chatzichristos, Pedro E. Alcaraz

**Affiliations:** 1 Faculty of Kinesiology, University of Zagreb, 10110 Zagreb, Croatia; igor.jukic@kif.unizg.hr (I.J.); luka.milanovic@kif.unizg.hr (L.M.); ivankrakan@gmail.com (I.K.); 2 Biotrenning Ltd., 10000 Zagreb, Croatia; 3 Faculty of Education and Sport, University of Basque Country, 01007 Vitoria-Gasteiz, Spain; 4 Strength and Conditioning Society, 00118 Rome, Italy; cosfrancesc@gmail.com (F.C.); palcaraz@ucam.edu (P.E.A.); 5 National Institute of Physical Education (INEFC), University of Barcelona, 08038 Barcelona, Spain; 6 Technogym SpA, 47521 Cesena, Italy; fcuzzolin@technogym.com; 7 Football Science Institute, 18016 Granada, Spain; jesusolmo@me.com (J.O.); bernardorequena@footballscienceinstitute.com (B.R.); 8 Unidad Regional de Medicina Deportiva, Avilés and Instituto de Investigación Sanitaria del Principado de Asturias (ISPA), 33401 Oviedo, Spain; nterrados@ayto-aviles.es; 9 Football Club Deportivo Alavés, 01007 Vitoria-Gasteiz, Spain; Nenad.Njaradi@gmail.com; 10 Football Club Juventus, 10151 Torino, Italy; r.sassi@gmail.com; 11 Basketball Club CSKA, Moscow 125167, Russia; kostas.chatz@gmail.com; 12 Research Center for High Performance Sport, UCAM, 30107 Murcia, Spain

**Keywords:** strategies, team sports, athletes, training, isolation, COVID-19

## Abstract

In December of 2019, there was an outbreak of a severe acute respiratory syndrome caused by the Coronavirus 2 (SARS-CoV-2 or COVID-19) in China. The virus rapidly spread into the whole World causing an unprecedented pandemic and forcing governments to impose a global quarantine, entering an extreme unknown situation. The organizational consequences of quarantine/isolation are: absence of organized training and competition, lack of communication among athletes and coaches, inability to move freely, lack of adequate sunlight exposure, inappropriate training conditions. Based on the current scientific, we strongly recommend encouraging the athlete to reset their mindset to understand quarantine as an opportunity for development, organizing appropriate guidance, educating and encourage athletes to apply appropriate preventive behavior and hygiene measures to promote immunity and ensuring good living isolation conditions. The athlete’s living space should be equipped with cardio and resistance training equipment (portable bicycle or rowing ergometer). Some forms of body mass resistance circuit-based training could promote aerobic adaptation. Sports skills training should be organized based on the athlete’s needs. Personalized conditioning training should be carried out with emphasis on neuromuscular performance. Athletes should also be educated about nutrition (Vitamin D and proteins) and hydration. Strategies should be developed to control body composition. Mental fatigue should be anticipated and mental controlled. Adequate methods of recovery should be provided. Daily monitoring should be established. This is an ideal situation in which to rethink personal life, understanding the situation, that can be promoted in these difficult times that affect practically the whole world.

## 1. Introduction

In December of 2019, there was an outbreak of a severe acute respiratory syndrome caused by the Coronavirus 2 (SARS-CoV-2 or COVID-19) in Wuhan, Hubei Province, China. The virus rapidly spread across the country and then into the whole world [1], causing an unprecedented pandemic [2], forcing governments to impose an almost global quarantine. At the beginning of 2020 (January–March), the whole world, including the world of sports, entered an extreme and unknown situation [2], where, gradually, all sports competitions were postponed and any organized training or practice was banned [3]. The health of the athletes, coaches and spectators became a priority. The major local and international competitions, such as the European Football Championship and the Olympic Games in Tokyo, were postponed for a year. This unusual global crisis has caused a major organizational, financial and social disruption to athletes, coaches, clubs and sports federations. All teams have allowed their athletes to return home, where they are in mandatory home isolation following government guidelines. Isolation, of course, does not allow athletes to follow their usual training and competition schedule. Regardless of duration, isolation could have a significant impact on the physical and mental state of an athlete. However, to the best of our knowledge, no previous evidence on this particular topic has been published.

The organizational consequences of quarantine/isolation are: absence of organized training and competition, lack of adequate communication between athletes and coaches, inability to move freely, lack of adequate sunlight exposure, inappropriate training conditions [4,5]. Staying in quarantine can have negative effects, not only on most physiological systems, but also in the players’ lives. For example, isolation at home can lead to poor and inappropriate nutrition, poor quality of sleep, addictions, loneliness, just to name a few negative lifestyle changes. The physiological adverse effects of isolation include an increase in body fat content and a decrease in muscle mass, impaired immunity, loss of mental sharpness and toughness, insomnia and depression [4,6]. All of these consequences can have both a short- and long-term negative effect on the athletes’ physical fitness and competitive performance. Although it is difficult to predict the duration of the global COVID-19 crisis at this time, it is possible to predict the loss of training-induced adaptation [7,8,9,10,11].

Therefore, first, it is extremely important to identify these effects and to understand the mechanisms and effects on all physiological systems, as well as their impact on athletic performance. Second, but no less important, it’s important to provide practical recommendations to coaches and athletes to reduce the unwanted consequences of the forced quarantine.

## 2. Detraining Effects in Isolation

The principle of training reversibility states that stop or markedly reduce training induces a partial or complete reversal of the previous developed adaptations, thus compromising athletic performance [12]. The reversibility principle is also known as detraining. The concept of detraining refers to the total or partial loss of the training-induced adaptation achieved through training [10]. Although athletes experience transition periods throughout their sports careers, usually coinciding with the end of their competition period, illness, injury, or other factors, the loss of physical activity is not comparable to the restriction that the current “stay at home” confinement represents. However, detraining is one of the biggest negative consequences of the forced quarantine.

Detraining affects different physiological systems (e.g., neuromuscular, cardiovascular, respiratory or muscle-skeletal) and their corresponding physical capacities (e.g., strength and power, endurance, speed or flexibility). Although some investigations have concluded that neural changes are long-lasting and did not affect the elements of H-reflex pathways [13], there is strong evidence to think the opposite. For example, it was reported that neuromuscular performance was impaired in top-level male kayakers after 5 weeks of either reduced training or complete training cessation [14]. A recent systematic review [15] revealed that the concurrent (CT) training-induced gains may be compromised with a short-term detraining period (2–4 weeks), leading to a return to baseline values. The authors also explained that a 4-week period of training cessation after CT with different resistances or aerobic training loads compromised training-induced gains in young men. They concluded that, despite scarce evidence, it seemed that regardless of the intensity of the previous endurance and resistance training during CT, only 2–4 weeks of training cessation can cause a significant and marked loss of performance. To date, the most used CT method is resistance circuit-based training (RCBT) [16]. RCBT is an effective training method for the concurrent development of maximum oxygen consumption (VO_2max_) and one repetition maximum (1-RM) bench press in healthy adults, independent of participant and load characteristics, as shown in the authors’ review and meta-analysis [16]. Therefore, some forms of home-based RCBT could easily be performed with simple equipment at home and promote both neuromuscular and metabolic adaptations, thus minimizing neuromuscular detraining effects [17].

Reductions in maximal and submaximal exercise performance occur within weeks after the cessation of training. These losses in aerobic performance decline cardiovascular function and muscle metabolic potential. Specifically, significant reductions in VO_2max_ have been described within 2 to 4 weeks of detraining [18]. The detraining effects were mainly: (1) an initial rapid decline in VO_2max_; (2) decrease in blood volume; (3) changes in cardiac hypertrophy; (4) decrease in the total hemoglobin content; (5) decreased skeletal muscle capillarization; and (6) disruption of temperature regulation. When absence of training continued beyond 2 to 4 weeks [18], the detraining effects became more severe. This results in: (1) further declines in VO_2max_; (2) reductions in maximal arterial-venous (mixed) oxygen difference; (3) changes in maximal oxygen delivery, which may result from decreases in total hemoglobin content and/or maximal muscle blood flow and vascular conductance; (4) declines in skeletal muscle oxidative enzyme activity; and (5) reductions in submaximal exercise performance, which may be related to changes in the mean transit time of blood flow through the active muscle and/or the thermoregulatory response (i.e., degree of thermal strain) to exercise. Therefore, athletes must incorporate some type of endurance exercise their daily routine to try and reverse some of the aforementioned effects of detraining. 

Flexibility is the ability to move a joint through its optimal range of motion. The ability to move a joint without restriction or pain depends on the condition of different structures, such as bone, muscle, and connective tissue. It also depends on the muscle’s ability to produce an adequate amount of force [19]. Decreases in flexibility have been reported after 8 weeks of detraining [20]. Given that the current isolation period could be longer than a month, it is recommended to incorporate exercises to maintain and improve flexibility. For example, neurodynamic treatments [21] or Tai chi, Ioga or Thai Chi may be a useful therapy for vestibular rehabilitation, improving dynamic balance control and flexibility [22]. 

Short-term detraining may specifically affect eccentric strength and the size of the Type II (FT: fast twitch) muscle fibers [23]. It has been suggested that performing eccentric muscle actions during training is essential to promote greater and longer-lasting neural adaptations to training [24] and that speed-strength is better maintained during periods of reduced training if previously the focus of training was on power development [25]. Loturco et al. [26] concluded that it may be important for coaches to include plyometric training, even in detraining periods, in order to avoid possible impairments in the Stretch-shortening function [26]. This simple advice could help in maintaining/improving all the neuromuscular indices relevant to athletes’ performance and could constitute the basis of an ideal detraining strategy in sports like track and field [26]. 

Reduced or complete absence of strength training can cause loss of muscle mass. Muscle atrophy results from an imbalance between protein degradation and synthesis in favor of the former [27]. When inactivity exceeds 4 weeks, there is a transition of FT fibers into Type I (ST: slow twitch), especially in sports, characterized by explosive actions, with the FT being more vulnerable to periods of inactivity than the ST type [28]. Although when training periods do not exceed two weeks, the changes in the distribution of muscle fibers are not noticeable in long distance runners or in strength and power athletes [10], after the first 15 days, there is a decrease in the transverse fibrillar area of approximately 0.6% per day [29]. This decrease in muscle size translates to a 7% and 12% reduction in strength and team sports athletes, after a period of inactivity ranging from 8 to 12 weeks. A decrease in FT fiber content has been observed in footballers and weightlifters [30] and a decrease in the ability to apply force to the water in swimmers [18]. Similarly, some fibrillar conversion of FTa fibers to FTx fibers has been observed in long-distance runners and cyclists [10]. 

**Figure 1:**
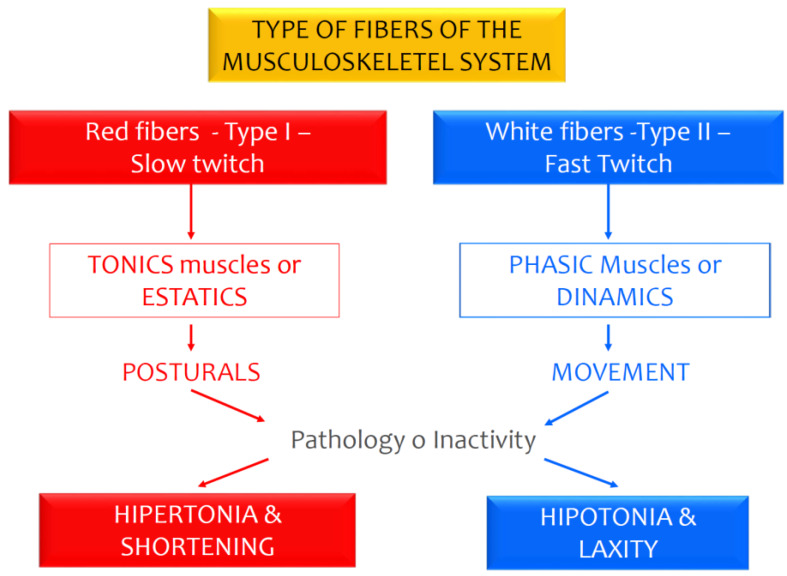
Fundamental characteristics of the tonic and phasic muscles, as well as the main physiological adaptations to pathology or inactivity. Characteristics and habitual response of the tonic and phasic muscles (Cos and Cos, 1999).

Periods of prolonged inactivity negatively affect the anti-gravitational muscle groups and the posterior extensor muscle chain [31]. In general, inactivity affects different muscles and muscle chains depending on whether they are tonic or phasic, causing muscle shortening and/or hypertonia or laxity and/or hypotonia depending on the muscle type (Figure 1). These imbalances can be the onset or worsening of pathologies such as groin pain [32]. Other authors hypothesized that inactivity also caused a decrease in collagen synthesis in the human tendon, with progressive decreases in collagen synthesis being recorded between 10 and 21 days of complete inactivity [33]. 

Reducation in activity results in a reduced energy expenditure, which consequently requires a reduction in energy intake to prevent unwanted body fat gains. In terms of an absolute amount of protein per day, when increasing protein to 2.3 g/kg, body mass reduces muscle loss during periods of reduce caloric intake [34]. Thus, athletes may benefit from increasing their protein intake to counter the immobilization-induced anabolic resistance, as well as to attenuate the associated losses in muscle mass [35]. It is accepted that when reducing energy intake, macronutrients should not be cut evenly, as maintaining a high-protein intake will be essential to attenuate loss in lean muscle mass. For instance, leucine consumption, which is a key and critical amino acid for stimulating the cell signaling pathways that control muscle protein synthesis, should be emphasized in the protein sources consumed [36].

## 3. Other Methodological Issues of Isolation

A major consideration when training athletes in home isolation is compliance, especially regarding the intensity and volume of exercise. It is difficult to monitor and ensure that the load that athletes use at home is appropriate to maintain physical fitness and performance at the required level. For recreational and ordinary people in isolation, maintaining an acceptable level of physical fitness is possible with moderate exercise [37], but high-level athletes need precise exercise prescription. Maintaining a high level of physical and mental fitness requires relatively high loads of submaximal and maximal intensity exercise [38]. Because of the lack of appropriate space (e.g., a football field) and the subsequent inability to perform sport-specific and/or high-intensity exercises, such as sprints, athletes returning to sport after the quarantine must be aware of an increased chance of injury. Therefore, sport governing bodies must offer appropriate time to the athletes and teams to prepare for high-level competition. 

Lack of competition poses an additional problem to teams and athletes, because it is through competitions that athletes can best maintain their physical fitness and sport form. Competing activities in many sports with a congested competition schedule [39] is also a key factor which is an important developmental stimulus [40]. In addition, preparatory, control and official competitions are an important tool to establish and maintain optimal performance [41]. Consequently, the absence of competition has a negative impact on athlete’s performance and peak sports form.

## 4. Window of Opportunity during the Isolation

In spite of everything, some positive effects of isolation should also be kept in mind. In such conditions, the athlete can fully recover from all stresses, injuries and previously accumulated loads (overreaching and overtraining). For example, in team sports there are very few situations in the regular annual calendar in which a player can have a prolonged period of complete recovery from specific training and competition demands. Only off-season/transition periods can offer some opportunity for rest [42]. Isolation and the absence of intensive specific training and competition enable both complete cellular recovery and the avoidance of common daily mental stress. This is also an opportunity to implement developmental programs of certain physical abilities for which an athlete in team sports does not have enough time under the regular periodization regimen [43]. Off-season/transition periods like this exceptional situation are also a rare opportunity to have enough time for extensive injury prevention and individual athletic development work. That work prepares athletes for a rushed pre-re-season, including high-intensity work in wide spaces, and good performance with low injury risk, when the competitions resume. A very similar situation occurs after an athlete suffers a serious injury. Those athletes who use rehabilitation as an opportunity for athletic development generally return to competition in a better shape for the rest of the season, which consequently positively affects their future career [44]. In other words, this isolation is an opportunity for both a complete physiological and mental reset as well as for the athlete’s integral development. All the previously mentioned training and recovery programs should be strictly personalized [45]. 

Based on the current scientific and practical evidence, we strongly recommend the following points:

- Encourage, provoke and motivate the athlete to reset their mindset and use this break as an opportunity for personal development [44];
- Organize appropriate guidance and support to athletes by experts (sports coach, strength and conditioning coach, nutritionist, doctor, psychologist) by using technology (video call, e-mail, telephone, text messages);
- Educate and encourage athletes to apply appropriate preventive behavior and hygiene measures to promote immunity and protect their own health and the health of the people in their immediate environment [6];
- Ensure good living conditions in isolation (space, equipment, food, telecommunications). If possible, the athlete’s living space should be equipped with cardio equipment (treadmill, bicycles, rowing ergometer, etc.), resistance training equipment (dumbbells, elastic bands, abdominal wheels, medicine balls, etc.) and other equipment for frequent use (mats, foam rollers, self-massagers, etc.). If not, some forms of body mass resistance circuit-based training could promote (or maintain) neuromuscular and aerobic adaptations [6];
- Organize alternative sports skills training (kinesthetic ball training in a small space, visualization, virtual reality technical aids, video analysis, theoretical training) based on the athlete’s deficits and needs;
- Organize personalized strength and conditioning training at home with available space and material resources that are tailored to the athlete’s individual characteristics and current needs [45]. Focus on neuromuscular plyometrics (i.e., vertical and horizontal jumping) and eccentric training (i.e., elastic bands), to maintain some key adaptations related to the stretch-shortening cycle, strength and power performance. Adaptations of the stabilizer muscles as an indispensable element and facilitator of the efficient sensorimotor action of any act is also extremely important [46,47];
- Educate the athlete about nutrition, supplementation (especially Vitamin D, zinc and proteins) and hydration in isolation conditions, and about strategies to control body mass and body composition [5,36,48,49]. It is important to consume food to fight off viral infections, thus advising against lower carbohydrate/intermittent fasting approaches is likely important [50];
- Organize mental fatigue monitoring and mental training (mental self-help techniques and/or the support of a psychologist by telecommunication) [51];
- Provide adequate methods of recovery (supplementation, sleep, breathing and meditation exercises, self-massage, myofascial relaxation, stretching, low back heat, etc.) [52];
- Use forms of self-assessment (heart rate monitoring, hearth rate variability, hearth rate recovery, orthostatic test, simple movement functional tests, simple VO2max tests, etc.) that an athlete can use on a daily basis and share data with a strength and conditioning coach [53];
- Establish daily monitoring of the athlete’s health, wellness, physical fitness, recovery [54] and workload by using technology (phone, applications, e-mail, text message) [55];
- Even though many athletes are not currently injured, the time off is similar to the time off after an injury [56];
- Finally, muscle memory is important to educate athletes, given that any losses are rapidly regained. This should quell some anxiety [57].

## 5. Conclusions

To conclude, an athlete’s life in isolation due to a COVID-19 crisis and imposed quarantine should have another, positive meaning. This is an ideal situation to rethink and reorganize one’s personal life and value system. Humility, gratitude, understanding of the global situation, empathy for other people, family values, attitude towards knowledge, spirituality, and helping the needy are just some of the values that can be promoted and truly lived in difficult times that affect practically the whole world. As athletes are often role models, in this challenging time they should take responsibility and promote “good values”. Thus, they can contribute to building better societies and better people and promote better human behavior.
